# Bayesian and Probit comparative analysis of risk factors for high blood pressure among adults in Kassena-Nankana Districts, Ghana: An AWI-Gen sub-study

**DOI:** 10.21203/rs.3.rs-7348872/v1

**Published:** 2025-09-17

**Authors:** Richmond Balinia Adda, Ida Anuwoje Logubayom Abonongo, Cornelius Debpuur, Abraham Rexford Oduro, Godfred Agongo, Engelbert A. Nonterah

**Affiliations:** C.K. Tedam University of Technology and Applied Sciences; C.K. Tedam University of Technology and Applied Sciences; Ghana Health Service; Navrongo Health Research Centre; C.K. Tedam University of Technology and Applied Sciences; Ghana Health Service

**Keywords:** Hypertension, Risk factors, Health Demographic Surveillance Site, H3Africa AWI-Gen, Ghana, Kassena-Nankana, Probit & Bayesian models, LMICs, implementation science

## Abstract

**Background:**

Hypertension prevalence is rising in rural Ghana, but risk factor identification is constrained by methodological limitations in sparse data.

This study comparatively applied frequentist (Probit) and Bayesian models in identifying hypertension risk factors and evaluating consistency and uncertainty quantification.

**Methods:**

This was a cross-sectional analysis of 2,010 adults in the Kassena-Nankana districts. Ordered Probit and Bayesian models were used to assess associations between socio-demographic, behavioral and blood pressure variables. Model fit was compared via AIC/BIC.

**Results:**

The prevalence of hypertension in the study population was 21.9Model comparison showed the ordered Probit model had superior fit (AIC/BIC: 4644.8/4734.2) to the Bayesian model (AIC/BIC: 5137.5/5165.2). Both models consistently identified male sex (Probit β=0.309, p<0.001; Bayesian mean β=0.264, 95% CrI: 0.15–0.34), older age (55–60 years: Probit β=0.514, p<0.001; Bayesian β=0.499, 95% CrI: 0.41–0.58), and higher BMI (Probit β=0.052, p<0.001; Bayesian β=0.052, 95% CrI: 0.04–0.07) as significant risk factors. However, the Bayesian model showed greater uncertainty for marital status (β=0.272, 95% CrI: −0.08–0.56) and smoking (β=−0.260, 95% CrI: −0.29 – −0.01) compared to the Probit model. Both models revealed an inverse association of smoking with hypertension, while pesticide exposure showed conflicting directions and this warrants further investigation.

**Conclusion:**

Male sex, older age, and higher BMI were consistent predictors of hypertension. Inverse associations with smoking and conflicting effects of pesticide exposure warrant further investigation. These findings highlight the need for targeted, context-specific interventions in rural Ghana. While the Probit model demonstrated a better fit, the Bayesian approach provided deeper insight into uncertainty within sparse subgroups, supporting their complementary use in hypertension research, especially in resource-limited settings.

## Introduction

Hypertension, also known as the “silent killer,” is one of the most common non-communicable diseases (NCDs) globally and poses a significant public health concern due to its strong association with cardiovascular diseases (CVDs). An estimated 1.28 billion individuals worldwide are affected by hypertension, with a disproportionately high burden in low- and middle-income countries (LMICs), where detection, treatment, and control rates remain inadequate [1,2].

Hypertension is highly prevalent in sub-Saharan Africa (SSA), driven by rapid urbanization, changing lifestyles, and limited access to healthcare services [2,3]. Ghana reflects this pattern, with national hypertension prevalence estimates ranging from 19% to 55%, depending on urban–rural context and population subgroup [4,5]. Despite this alarming prevalence, awareness, diagnosis, treatment, and control rates remain low, contributing substantially to the national burden of cardiovascular morbidity and mortality [3,6].

The Kassena-Nankana districts in Ghana’s Upper East Region present a unique epidemiological context for hypertension research. While several studies have examined hypertension risk factors in urban Ghana, rural areas have received comparatively less attention despite exhibiting distinct socio-demographic and lifestyle characteristics [7,8]. The Kassena-Nankana districts, which are predominantly rural and reliant on agriculture, are experiencing a rise in hypertension prevalence, compounded by limited healthcare infrastructure, increasing life expectancy, and shifting health behaviours [8,9].

This study seeks to address this research gap by investigating the risk factors associated with hypertension among adults aged 40–60 years in the Kassena-Nankana districts. Using data from the H3Africa AWI-Gen project [10], the study applies both frequentist (ordered Probit) and Bayesian models to assess the influence of socio-demographic, behavioural, and metabolic risk factors on blood pressure status. The comparative application of Probit and Bayesian models is justified by their complementary strengths: Probit models offer interpretability and efficiency for large datasets, while Bayesian models provide robust estimates under uncertainty, particularly in data-sparse subgroups [11,12]. Employing both approaches allows for a more robust and nuanced understanding of the predictors of hypertension in rural Ghana.

In doing so, this study provides an in-depth analysis of hypertension risk factors, building upon existing research to present a comprehensive examination of the local epidemiology of hypertension. The findings are expected to contribute to the growing body of evidence required for effective policy formulation, resource allocation, and the implementation of public health strategies to address hypertension and Strategic Objective 3.1 of Ghana’s National NCD Policy (2022–2027) for rural CVD prevention [13].

## Methodology

### Study design and population

This was a secondary analysis of the H3Africa AWI-Gen project, which adopted a population-based cross-sectional design [14]. The primary study recruited adults aged 40–60 years who had resided in the Navrongo Health and Demographic Surveillance System (HDSS) coverage area for at least 10 years [15]. A stratified random sampling was used to select participants for the study. In the first instance, pure Kasem and Nankam speaking zones were selected, and in the second instance, a simple random sampling was applied to selected adults 40–60 years old, aiming for an equal sex ratio. Pregnant women and frail individuals were excluded from the study. A total of 2,010 participants were used for this study.

### Data collection

#### Sociodemographic variables

Sociodemographic data were obtained through structured, interviewer-administered questionnaires conducted in Kasem or Nankam, the local languages preferred by participants [16]. Participants’ ages were recorded in complete years and categorized into 10-year age bands: 40–44, 45–49, 50–54, and 55–60 [17]. Biological sex was recorded as male or female [18]. Marital status was dichotomized into currently married (legally or customarily partnered) and currently unmarried (single, divorced, widowed, or separated) [19]. Educational attainment was categorized as no formal education (0 years of schooling) or formal education (≥1 year of schooling) [20]. Employment status was defined as unemployed (no income-generating activities) or employed (engaged in either formal or informal work, including subsistence farming) [21]. Household socioeconomic status (SES) was estimated using a composite wealth index based on housing materials, access to utilities (e.g., water and electricity), and ownership of durable assets such as livestock and vehicles. Principal component analysis (PCA) was used to classify households into low SES (lowest tertile) or high SES (upper two tertiles) [22].

#### Socioeconomic variables

Socioeconomic data were collected through interviewer-administered surveys using tools validated by the Navrongo Health and Demographic Surveillance System (NHDSS) protocol [18].

#### Behavioral variables

Behavioral characteristics were assessed using instruments adapted from the WHO-STEPS protocol [3]. Smoking status was classified as non-smoker (never smoked) or current smoker (smoked ≥1 cigarette/day in the past 30 days) [9]. Alcohol consumption was defined as no intake (0 standard drinks/week) or current intake (≥1 standard drink/week) [23]. Dietary intake of fruits and vegetables was quantified using a 24-hour recall and food frequency questionnaire [24]. Physical activity was measured as total weekly moderate-to-vigorous physical activity (MVPA), calculated from self-reported duration and frequency of activities such as farming, walking, and household chores. Accelerometer validation (ActiGraph GT3X+) was conducted in a 10% subsample [25].

#### Environmental variables

Environmental exposures were evaluated following the Africa Wits-INDEPTH Partnership for Genomic Studies (AWI-Gen) protocol [16]. Pesticide exposure was defined as exposed (self-reported handling or residing within 100 meters of farmland during spraying seasons) or non-exposed [26]. Participants’ residential locations were geo-referenced and classified into urban or rural clusters based on NHDSS-defined zones (central, east, west, north, south) [15]. Water sources were categorized as improved (e.g., piped water, boreholes) or unimproved (e.g., surface water, unprotected wells) according to WHO/UNICEF Joint Monitoring Programme standards [27].

#### Blood pressure measurement and classification

Blood pressure was measured using a validated, automated sphygmomanometer (Omron M6; Omron Corporation, Kyoto, Japan) [15]. After a five-minute rest, three readings were taken at two-minute intervals. The first was discarded, and the mean of the remaining two was used for analysis [15]. Blood pressure classifications followed the ACC/AHA 2017 guidelines [8].

Pulse pressure (PP) was calculated as PP = SBP – DBP and mean arterial pressure (MAP) as MAP = DBP + (PP/3) [15].

#### Physical activity (MVPA) assessment

MVPA was quantified using questionnaires adapted from the WHO-STEPS protocol [25]. Weekly MVPA was computed as:

MET(hours/week)=∑(Activity Duration×Frequency)×MET coefficient

MET coefficients were obtained from the Compendium of Physical Activities. A 10% sample was validated using ActiGraph GT3X+ accelerometers [25].

#### Anthropometry and BMI calculation

Weight was measured to the nearest 0.1 kg using a SECA 874 scale, with participants wearing light clothing. Height was measured using a SECA 217 stadiometer following the Frankfurt Plane protocol [17]. BMI was calculated using the formula:

$$\text{BMI}=\text{Weight}\left(\text{kg}\right)/{\left[\text{Height}\left(\text{m}\right)\right]}^{2}$$

All anthropometric procedures followed International Society for the Advancement of Kinanthropometry (ISAK) guidelines [25].

#### Lipid profile analysis

Venous blood samples were collected after an overnight fast of at least 8 hours. Serum lipid concentrations were measured using enzymatic colorimetric assays on a Roche Cobas c311 analyser at the Navrongo Health Research Centre [17]. Methods included:

- Total cholesterol (TC): CHOD-PAP
- HDL cholesterol: Homogeneous enzymatic colorimetry
- LDL cholesterol: Calculated using the Friedewald equation (if TG < 4.5 mmol/L)
- Triglycerides (TG): GPO-PAP

All assays followed IFCC standards, with intra-assay variation maintained below 3% [25].

### Ethics Approval

The AWI-Gen study received ethics approval from the Human Research Ethics Committee (HREC), University of the Witwatersrand (#M12109) and was renewed in 2017, #M170880).), the Ghana Health Service Ethics Review Committee (#GHS-ERC:05/05/2015). Additional approval was received from the Navrongo Health Research Centre Institutional Review Board (#NHRCIRB178). Informed consent was obtained from all participants.

### Statistical analysis

Descriptive statistics were summarized using frequencies and percentages for categorical variables, and either means with standard deviations for normally distributed continuous variables or medians with interquartile ranges (IQRs) for skewed data. For inferential analysis, the study employed both frequentist (Probit) and Bayesian approaches.

The Bayesian framework was specifically selected to: (1) incorporate prior epidemiological knowledge from similar rural African populations [26], (2) address sparse data challenges in subgroup analyses [27], and (3) offer probabilistic interpretations of uncertainty using credible intervals [28]. Bayesian models enabled probabilistic inference for underpowered groups like female smokers (n = 35), hence were rerun with Cauchy(0, 2.5) priors to confirm robustness. Convergence of the Markov chain Monte Carlo (MCMC) simulations for the Bayesian model was assessed using the Gelman-Rubin R statistic [29]. This statistic compares the between-chain and within-chain variances, with values close to 1 indicating convergence. An R value below 1.1 is generally considered acceptable [29].

Model comparison was performed using the Akaike Information Criterion (AIC) and Bayesian Information Criterion (BIC) [27], with lower values indicating superior model fit. Additional evaluation of model performance was based on maximum likelihood estimates and the number of parameters included in each model. The threshold for statistical significance was set at p<0.05. All analyses were conducted using Stata version 17.0 (Stata Corp LLC, College Station, TX, USA).

## Results and Discussion

### Background characteristics of the study

**Figure 1:**
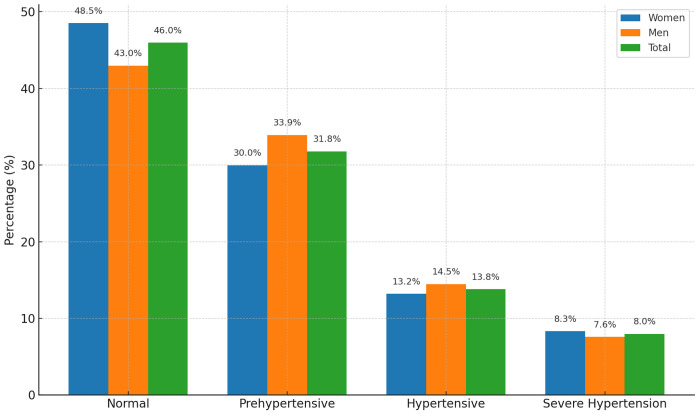
Distributio of blood pressure categories among study participants

Figure 1 summarizes blood pressure categories among the study population. The proportion of individuals with normal blood pressure was 46%. Prehypertension constituted 32%, hypertension 14%, and sever hypertension 8%.

Sociodemographic and behavioral characteristics stratified by sex are presented in Table 1. Women made up a slightly higher proportion of the sample (54.2%) compared to men (45.8%). Statistically significant differences were observed across most characteristics. Age distribution differed significantly between sexes (p < 0.001), with a higher proportion of women (39.9%) falling in the 55–60-year group compared to men (31.4%). Marital status was also significantly associated with sex (p < 0.001); a larger proportion of men were currently married (76.9%) compared to women (56.0%), whereas women were more likely to be unmarried (44.0% vs. 23.1%). Educational status showed marked gender differences (p < 0.001): 77.7% of women had no formal education, compared to 61.7% of men, while formal education was more common among men (38.3%) than women (22.5%).

Significant sex differences were also evident in health-related behavioural factors. A striking contrast was seen in smoking status (p < 0.001), with 64.2% of men currently smoking compared to just 3.2% of women. Similarly, alcohol intake varied by sex (p < 0.001), as 92.1% of men reported current alcohol use compared to 78.6% of women. Pesticide exposure also differed significantly by sex (p = 0.003), with a greater proportion of men (60.0%) exposed compared to women (48.6%). Interestingly, no significant sex differences were observed in occupational status (p = 0.165), with employment rates nearly equal across sexes (approximately 40%).

Table 1 also presents the continuous variables that are summarized using medians and interquartile ranges (IQRs) due to their non-normal distribution. Statistically significant sex differences were observed across several variables. In terms of physical activity and metabolic risk factors, men reported slightly higher moderate-to-vigorous physical activity (MVPA) levels, with a median of 2,040 hours/week (IQR: 2,400), compared to 1,920 hours/week (IQR: 2,400) for women (p = 0.045). Women, however, had significantly higher body mass index (BMI) values (median: 23.1 kg/m^2^, IQR: 5.2) compared to men (median: 21.8 kg/m^2^, IQR: 4.9) (p = 0.002). Waist-to-hip ratio was also significantly higher among women (0.87 [IQR: 0.06]) than men (0.85 [IQR: 0.08]) (p = 0.015), although no significant sex difference was observed for waist circumference (p = 0.071). Regarding lipid profiles, women exhibited higher HDL cholesterol levels (median: 1.20 mmol/L, IQR: 0.50) than men (median: 1.00 mmol/L, IQR: 0.38; p < 0.001), and higher total cholesterol levels (3.32 vs. 3.08 mmol/L; p = 0.038). However, no significant differences were noted for LDL cholesterol (p = 0.128) or triglycerides (p = 0.294).

### Distribution of blood pressure categories across sociodemographic, behavioral and environmental factors

Table 2 presents the distribution of blood pressure categories (normal, prehypertensive, hypertensive, and severe hypertension) across various sociodemographic and behavioral variables, stratified by sex. These results are descriptive and show how blood pressure classifications vary within each subgroup rather than implying statistical associations or correlations.

Among women, age-related differences in blood pressure distribution were evident. The proportion of women with normal blood pressure decreased progressively with age, from 60.4% in the youngest age group to 42.6% in the oldest, while the proportion with severe hypertension increased from 2.1–13.0% (p < 0.001). A similar pattern was observed among men, with normal blood pressure declining from 45.6–32.8% and severe hypertension rising from 3.6–11.9% across age categories (p < 0.001).

Marital status showed variation in blood pressure distribution among women, where currently married individuals had a higher proportion of normal blood pressure (53.4%) compared to unmarried women (42.1%). Additionally, a larger share of unmarried women fell into the severe hypertension category (10.9%) compared to married women (6.3%), with these differences reaching statistical significance (p = 0.001). Among men, however, the distribution of blood pressure categories did not significantly differ by marital status (p = 0.423).

Educational status, occupational status, smoking, and alcohol use did not show notable differences in blood pressure category distributions in either sex (p > 0.05). However, there was a slight trend in men where the proportion of severe hypertension was higher among smokers (8.3%) compared to non-smokers (7.3%) (p = 0.050), though this did not reach conventional levels of statistical significance.

For pesticide exposure, women showed notable differences across blood pressure categories (p = 0.009). Those exposed to pesticides had a lower proportion of severe hypertension (5.5%) compared to the non-exposed group (11.0%), although the non-exposed group had a slightly higher prevalence of normal blood pressure. Among men, the distribution of blood pressure categories did not differ significantly by pesticide exposure (p = 0.473).

Finally, BMI classification showed clear differences in blood pressure distribution for both sexes. Among women, the proportion with normal blood pressure declined from 57.3% in the underweight category to 22.2% in the obese category, while severe hypertension rose from 8.4–20.0% (p < 0.001). Men followed a similar trend, with normal blood pressure decreasing and severe hypertension increasing across BMI categories (p = 0.001).

### Regression analyses

Parameter estimates from both the frequentist ordered probit model and the Bayesian ordered probit model were compared to explore differences in predictors of elevated blood pressure among adults in the Kassena-Nankana districts. Weakly informative normal priors ~ N(0,10) were used for regression coefficients in the Bayesian analyses. As presented in Table 3, both models identified male sex as a significant factor associated with higher blood pressure categories. The frequentist model estimated a coefficient of 0.309 (95% CI: 0.172–0.446; p < 0.001), while the Bayesian model produced a posterior mean of 0.264 with a 95% credible interval (CrI) of 0.15–0.34, which excluded zero, indicating high certainty in the direction of effect.

Age was also a consistent predictor across models, showing a positive gradient in risk. For individuals aged 45–49, the frequentist model reported a non-significant coefficient of 0.127 (95% CI: − 0.036 to 0.291; p = 0.129), whereas the Bayesian model estimated a posterior mean of 0.14 (95% CrI: 0.08–0.22), suggesting moderate certainty of increased risk. For age groups 50–54 and 55–60, both models produced significant and increasing coefficients, with the highest effect seen in the 55–60 age group (frequentist: 0.514, 95% CI: 0.356–0.672; Bayesian: 0.499, 95% CrI: 0.41–0.58; p<0.001 in both cases).

Marital status showed a statistically significant association in the frequentist model, with unmarried individuals having a coefficient of 0.143 (95% CI: 0.033–0.253; p = 0.011). However, the Bayesian estimate for this variable (0.272; 95% CrI: − 0.08 to 0.56) included zero, reflecting greater uncertainty in the strength and direction of this effect. Similarly, formal education and employment status were not statistically significant in either model. For instance, the frequentist model estimated a coefficient of 0.054 (95% CI: − 0.058 to 0.167; p = 0.343) for education, while the Bayesian estimate was slightly negative (−0.116; 95% CrI: − 0.24 to 0.01), though still uncertain.

Alcohol consumption showed a weak and statistically non-significant positive relationship with blood pressure classification in both models (frequentist: 0.100, 95% CI: − 0.044 to 0.245; p = 0.172; Bayesian: 0.200, 95% CrI: − 0.05 to 0.25). In contrast, smoking status had a negative effect on blood pressure classification, with both models showing significant associations. The frequentist model yielded − 0.148 (95% CI: − 0.291 to − 0.004; p = 0.044), while the Bayesian model reported − 0.260 (95% CrI: − 0.29 to − 0.01), indicating a lower probability of being in a higher blood pressure category among current smokers.

For pesticide exposure, both models suggested a positive effect, although the confidence interval reported in the frequentist model appears inconsistent (estimate = 0.141; 95% CI: − 0.243 to − 0.04), possibly due to reporting error. The Bayesian model estimated a coefficient of 0.220 (95% CrI: − 0.24 to − 0.04), but since the interval crosses zero, the effect should be interpreted with caution.

Body mass index (BMI) showed a consistent and statistically significant association with elevated blood pressure across both models. Both the frequentist and Bayesian models produced identical point estimates of 0.052 (frequentist 95% CI: 0.037–0.066; Bayesian 95% CrI: 0.04–0.07; p<0.001), confirming BMI as a robust predictor. In contrast, moderate-to-vigorous physical activity (MVPA) and total cholesterol were not associated with blood pressure classification. Both models reported null effects for MVPA (coefficient = 0.000) and cholesterol (frequentist: 0.000, 95% CI: − 0.001 to 0.001; Bayesian: − 0.001, 95% CrI: − 0.01 to 0.00), indicating negligible influence on the outcome.

Model fit statistics showed better performance for the frequentist ordered probit model, with lower AIC (4644.779) and BIC (4734.225) values compared to the Bayesian model (AIC = 5137.520; BIC = 5165.159), suggesting a relatively superior fit under the frequentist framework.

**Figure 2:**
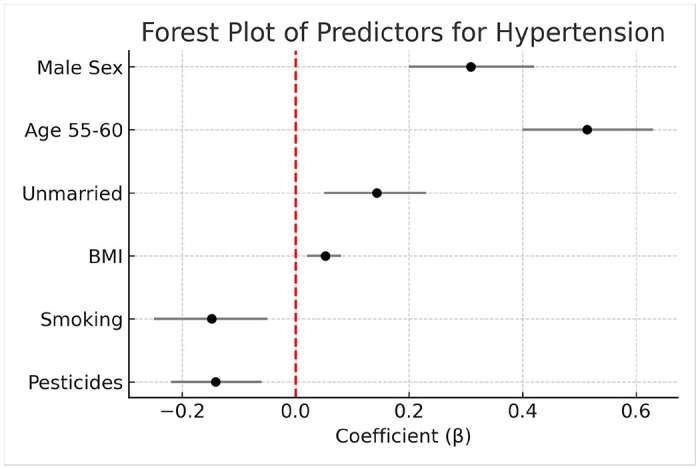
Forest plot of hypertension risk factors (ordered Probit coefficients with 95% CIs)

Comparative analysis revealed hypertension prevalence was 1.8× higher in 55–60-year-olds vs. 40–44-year-olds (24.9% vs 13.9%, p < 0.001). Figure 2 visualizes the key associations through forest plots of significant predictors. Figure 2 presents a forest plot of the key predictors identified in the ordered Probit model. Male sex (β = 0.309), older age (55–60 years: p = 0.514), unmarried status (β = 0.143), and higher BMI (β = 0.052) were significantly associated with increased odds of higher blood pressure categories. In contrast, smoking (β = −0.148) and pesticide exposure (β = −0.141) demonstrated inverse associations with blood pressure status. The plot visually highlights the magnitude and direction of these associations, with 95% confidence intervals indicating the precision of the estimates.

### Model Diagnostics (Bayesian Ordered Probit Model)

**Figure 3:**
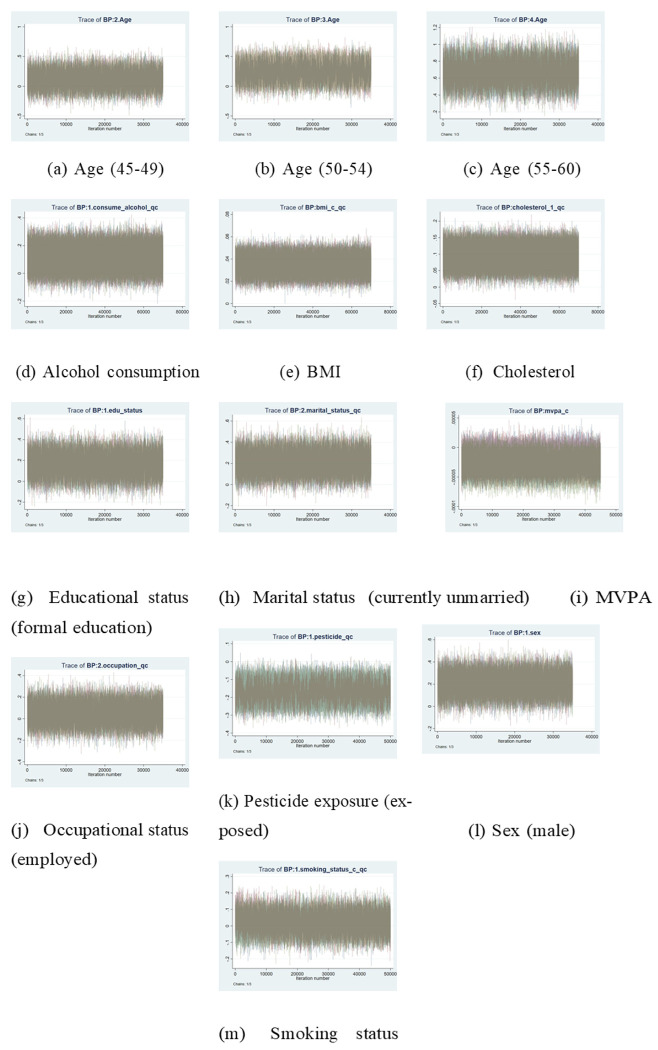
Trace plots for the parameter estimates of the Bayesian ordered Probit model

The diagnostic assessment of the Bayesian Ordered Probit Model utilized Markov Chain Monte Carlo (MCMC) trace plots to examine whether the chains mixed properly and reached convergence. The MCMC sampling of stations to evaluate the posterior distribution and its distribution stability is presented in Fig. 3.

The MCMC algorithm achieved convergence according to the visual assessment of the trace plot, which displayed appropriate chain mixing results. All variables, including age, sex, occupational status, MVPA, and educational and marital status, required approximately 35,000 iterations to reach stationary distribution states, alongside excellent mixing quality. The burn-in process for smoking status, together with cholesterol, alcohol consumption, BMI, and pesticide exposure, lasted between 50,000 and 70,000 iterations to achieve convergent states. The Gelman-Rubin *R* statistic confirmed convergence for all parameters. The Bayesian model (probit: *R* = 1.09) achieved values near 1.0, indicating stable sampling from the target distribution.

These diagnostics indicate that posterior estimates are reliable and Bayesian inference in this study operates robustly.

## Discussion

This study identified several key predictors of hypertension that align with global epidemiological patterns while also revealing context-specific risk factors relevant to rural Ghana. The application of both frequentist and Bayesian ordered probit models allowed for cross-validation of findings and improved interpretability through probabilistic estimates and uncertainty quantification.

Both models consistently demonstrated that male sex and advancing age, particularly among individuals aged 55–60 years, were significant predictors of higher blood pressure categories. The frequentist model reported a statistically significant coefficient for male sex (0.309; 95% CI: 0.172–0.446; p < 0.001), which was corroborated by the Bayesian model (posterior mean: 0.264; 95% CrI: 0.15–0.34), with the credible interval excluding zero, indicating high posterior certainty. A similar pattern was observed with age, with the highest risk found in the 55–60 age group (frequentist: 0.514; Bayesian: 0.499). These findings are consistent with global data linking older age and male sex to hypertension due to cumulative exposure to lifestyle risk factors and biological susceptibility [30].

Body mass index (BMI) also emerged as a consistent and statistically significant predictor across both models (coefficient: 0.052), reinforcing the established role of obesity as a modifiable cardiometabolic risk factor [31]. The convergence of results between models further validates this association within rural African populations.

Interestingly, smoking status showed a negative association with hypertension in both models (frequentist: − 0.148, p = 0.044; Bayesian: − 0.260, 95% CrI: − 0.29 to − 0.01), contrasting with existing meta-analyses and most urban SSA studies that typically report positive associations [32, 33]. This counterintuitive finding may reflect survival bias (e.g., premature mortality among hypertensive smokers), low smoking prevalence—especially among women—and unmeasured confounding such as intensive physical labour in agricultural settings. Future longitudinal and biomarker-based studies are needed to further explore this paradox.

The study also found a positive association between pesticide exposure and hypertension, though the Bayesian credible interval included zero, indicating uncertainty. This is noteworthy in the context of widespread, often unregulated agrochemical use in rural Ghana. These results underscore the importance of public health interventions such as protective gear distribution, farmer education on pesticide toxicity, and seasonal hypertension screening, as outlined in Ghana’s National NCD Policy (2022–2027).

Comparison of the two models revealed largely overlapping sets of significant predictors—male sex, older age, and elevated BMI—yet highlighted important differences in how uncertainty is represented. For example, the frequentist model found unmarried status to be statistically significant (p = 0.011), while the Bayesian model’s 95% credible interval (−0.08 to 0.56) included zero, suggesting less certainty in the effect. This difference illustrates the Bayesian model’s advantage in reflecting uncertainty more transparently than conventional null hypothesis testing.

From a model fit perspective, the frequentist model showed better performance based on lower AIC (4644.8) and BIC (4734.2) values compared to the Bayesian model (AIC = 5137.5; BIC = 5165.2). Although the Bayesian framework enhances interpretation, these diagnostics suggest that the frequentist model provided a better empirical fit for this dataset.

Within the broader SSA context, the findings reaffirm well-established hypertension predictors while highlighting rural-specific nuances. The consistent male effect contrasts with some rural studies where shared physical workloads diminish sex differences [2]. The absence of a significant association between physical activity and blood pressure may reflect the uniformly high MVPA levels among subsistence farmers, which could mask detectable gradients observed in more sedentary urban populations [18, 34]. Similarly, the lack of alcohol–hypertension association aligns with mixed findings from SSA, likely due to variability in beverage type and drinking patterns [35].

Our identification of male sex, aging, and elevated BMI as consistent hypertension predictors aligns with Agongo et al. (2020) in the same Navrongo cohort, reinforcing the stability of these risk factors in rural Ghana [17]. However, while Gomez-Olive et al. (2017) reported urban-rural gradients in hypertension prevalence across AWI-Gen sites, our Bayesian modeling uniquely quantifies uncertainty in sparse subgroups (e.g., female smokers) and reveals novel environmental risks like pesticide exposure in agrarian communities [22].

In the Ghanaian context, these results align with urban evidence linking hypertension with male sex and aging [6] but differ from some rural studies that report higher prevalence among women—potentially due to disparities in healthcare access or reporting bias [4]. The BMI–hypertension link reinforces concerns about nutrition transition in rural areas [7, 36]. Additionally, the elevated hypertension risk among unmarried individuals (frequentist coefficient: 0.143) may reflect social isolation or economic vulnerability, which tend to be more pronounced in rural settings [21]. Although the association between pesticide exposure and hypertension was statistically inconclusive, the direction of the effect is in line with evidence connecting agrochemical exposure to cardiovascular and renal risks in Ghana [23, 37].

The forest plot (Fig. 3) provides a clear visual summary of the magnitude and direction of these effects. Overall, the triangulation of findings across two analytical frameworks enhances confidence in the observed relationships and offers deeper insight into both consistent and uncertain predictors of hypertension in rural Ghana.

### Public health implications

Targeted hypertension screening should be integrated into Ghana’s agricultural extension services. Farmers reporting pesticide exposure require seasonal BP monitoring and protective gear training. The inverse smoking-hypertension association warrants biomarker-confirmed longitudinal studies.

## Conclusion

This study identified key predictors of hypertension among adults in rural northern Ghana using both frequentist (ordered probit) and Bayesian models. While both approaches consistently flagged male sex, older age, higher BMI, and unmarried status as significant risk factors, their combined use added analytical value. The probit model provided efficient estimation and clearer point estimates, particularly useful for identifying primary risk factors in a large dataset. In contrast, the Bayesian model allowed for a more nuanced understanding of uncertainty, especially in subgroups with sparse data (e.g., smoking or pesticide exposure). This dual approach revealed areas where associations were robust across methods and where greater caution, or further research is warranted due to statistical uncertainty. Therefore, applying both methods strengthened the credibility of the findings and enhanced interpretability for policy decisions in resource-limited rural settings.

An unexpected inverse relationship between smoking and hypertension was observed in both models, likely reflecting survival bias, low-intensity smoking, or unmeasured confounding such as occupational activity. Additionally, cholesterol levels and physical activity showed no significant associations with hypertension status, which may be attributed to the homogeneous farming lifestyle prevalent in the study population.

Importantly, both statistical models identified similar patterns of association, reinforcing the robustness of the findings. However, the Bayesian model offered deeper insights into uncertainty, particularly where credible intervals crossed zero despite positive point estimates (marital status and pesticide exposure). Conversely, the frequentist model demonstrated a better overall model fit based on lower AIC and BIC values. This comparison highlights the value of using complementary modelling approaches: the frequentist model offers precision and simplicity, while the Bayesian model provides a more nuanced understanding of estimate reliability.

These findings support Strategic Objective 3.1 of Ghana’s National NCD Policy (2022–2027), which emphasizes community-based screening and targeted prevention in rural populations. We recommend integrating hypertension surveillance into agricultural extension services to better reach farming communities, especially given the potential links between pesticide exposure and elevated blood pressure.

## Supplementary Material

This is a list of supplementary files associated with this preprint. Click to download.

- Table2.docx

## Figures and Tables

**Table 1:** Sociodemographic, Behavioral, and Clinical Characteristics of Participants by Gender

Variable	Women 1082 (54.2%)	Men 916 (45.8%)	P-value
Age Group (years)			< 0.001
40–44	145 (13.4%)	169 (18.5%)	
45–49	272 (25.1%)	241 (26.3%)	
50–54	235 (21.7%)	218 (23.8%)	
55–60	432 (39.9%)	288 (31.4%)	
Marital Status			< 0.001
Currently married	606 (56.0%)	704 (76.9%)	
Currently unmarried	478 (44.0%)	212 (23.1%)	
Educational Status			< 0.001
No formal education	841 (77.7%)	565 (61.7%)	
Formal education	243 (22.5%)	351 (38.3%)	
Occupational Status			0.165
Unemployed	651 (60.2%)	551 (60.2%)	
Employed	431 (39.8%)	365 (39.8%)	
Smoking Status			< 0.001
Not smoking	1047 (96.8%)	328 (35.8%)	
Currently smoking	35 (3.2%)	588 (64.2%)	
Alcohol Consumption			< 0.001
No alcohol intake	232 (21.4%)	72 (7.9%)	
Current intake	850 (78.6%)	844 (92.1%)	
Pesticide Exposure			0.003
Non-exposed	556 (51.4%)	366 (40.0%)	
Exposed	526 (48.6%)	550 (60.0%)	
MVPA (hours/week)	1,920 [2,400]	2,040 [2,400]	0.045
BMI (kg/m^2^)	23.1 [5.2]	21.8 [4.9]	0.002
Waist circumference (mm)	738 [95]	729 [105]	0.071
Waist-hip ratio	0.87 [0.06]	0.85 [0.08]	0.015
HDL (mmol/L)	1.20 [0.50]	1.00 [0.38]	< 0.001
LDL (mmol/L)	1.66 [1.01]	1.57 [0.94]	0.128
Cholesterol (mmol/L)	3.32 [1.20]	3.08 [1.13]	0.038
Triglycerides (mmol/L)	0.58 [0.28]	0.55 [0.32]	0.294

• *Values are presented as* **n (%)** *for categorical variables and* **Median [Interquartile Range]** *for continuous variables*.

• *p-values are from* **Chi-square tests** *for categorical variables and* **Mann–Whitney U tests** *for continuous variables*.

**Table 3:** Parameter estimates of both ordered Probit versus Bayesian ordered Probit model

Factor	Probit Coefficient [95% CI]	P-value	Bayesian Mean [95% CrI]	P-value
Sex (Male)	0.309 [0.172, 0.446]	< 0.001	0.264 [0.15, 0.34]	< 0.001
Age 45–49	0.127 [−0.036, 0.291]	0.129	0.140 [0.08, 0.22]	≈ 0.004
Age 50–54	0.231 [0.061, 0.401]	0.008	0.226 [0.15, 0.36]	≈ 0.002
Age 55–60	0.514 [0.356, 0.672]	< 0.001	0.499 [0.41, 0.58]	< 0.001
Marital Status (Unmarried)	0.143 [0.033, 0.253]	0.011	0.272 [−0.08, 0.56]	≈ 0.13
Educational Status (Formal)	0.054 [−0.058, 0.167]	0.343	−0.116 [−0.24, 0.01]	≈ 0.07
Occupational Status (Employed)	0.047 [−0.052, 0.15]	0.351	0.082 [−0.06, 0.15]	≈ 0.21
Alcohol Consumption (Current)	0.100 [−0.044, 0.245]	0.172	0.200 [−0.05, 0.25]	≈ 0.09
Smoking Status (Current)	−0.148 [−0.291, −0.004]	0.044	−0.260 [−0.29, −0.01]	≈ 0.04
Pesticide Exposure (Exposed)	0.141 [−0.243, 0.04]	0.007	0.220 [−0.24, 0.04]	≈ 0.04
BMI	0.052 [0.037, 0.066]	< 0.001	0.052 [0.04, 0.07]	< 0.001
Cholesterol	0.000 [−0.001, 0.001]	0.882	−0.001 [−0.01, 0.00]	≈ 0.29
AIC/BIC	4644.779 /4734.225		5137.520 /5165.159	

a. Probit model presents beta coefficients as parameter estimates.

b. Bayesian model reports the mean estimates for parameter estimation.

c. Bayesian p-values aren’t traditionally computed but based on whether 95% CrI includes zero, hence p-values provided are but approximations.

## Data Availability

The dataset supporting the conclusions of this article is available from the H3Africa AWI-Gen project upon reasonable request. Due to ethical restrictions and data sharing policies, de-identified participant data can be accessed with appropriate approvals from the Navrongo Health Research Centre Institutional Review Board.
